# Industrial Bread Composition: Potential Implications for Patients with Inflammatory Bowel Disease

**DOI:** 10.3390/nu17132120

**Published:** 2025-06-26

**Authors:** Shelly Shakhman, Tamar Pfeffer-Gik, Sarine Elial-Fatal, Yelena Broitman, Henit Yanai, Uri Gophna, Iris Dotan, Lihi Godny

**Affiliations:** 1 Division of Gastroenterology, Rabin Medical Center, Petah Tikva 4941492, Israel; shellyshk@gmail.com (S.S.);; 2 Nutrition Department, Rabin Medical Center, Petah Tikva 4941492, Israel; 3 Faculty of Medical and Health Sciences, Tel Aviv University, Tel Aviv 6997801, Israel; 4 The Shmunis School of Biomedicine and Cancer Research, Tel Aviv University, Tel Aviv 6997801, Israel

**Keywords:** inflammatory bowel disease (IBD), ultra-processed foods (UPFs), food additives (FAs), microbiome, emulsifiers, preservatives

## Abstract

**Background**: Ultra-processed food (UPF) intake, particularly that of industrial breads rich in food additives (FAs) like emulsifiers, has been linked to higher risk of inflammatory bowel diseases (IBD). Here, we screened the ingredients and FAs used in the bread industry and reviewed their potential biological effects. **Methods**: We consecutively screened breads available at supermarket and health food store chains in Israel. Bread products were analyzed by dietitians and categorized into three categories based on their composition: low processed (traditional ingredients), medium processed (additives like malt and fibers), and highly processed (FAs like emulsifiers and preservatives). We conducted a literature review to explore the links between the identified FAs, microbial composition and intestinal inflammation. **Results**: Of the 233 breads screened, 195 (84%) were highly processed, 9 (4%) medium-processed and 29 (12%) low-processed. We identified 37 different FAs and ingredients used. Most breads contained preservatives—189 (81%), and emulsifiers—178 (76%). Calcium propionate (E-282) was the most prevalent preservative present in 112 (48%) breads, while sodium-stearoyl-2-lactylate (SSL-E-481) was the most prevalent emulsifier present in 86 (37%) breads. The literature review revealed that 19 (51%) FAs used in the bread industry were associated with the exacerbation of inflammation or gut microbiome dysbiosis by increasing cytokine production and adversely affecting microbial composition. **Conclusions**: Most of the available breads in Israel are highly processed, containing FAs that may mediate intestinal inflammation. Low-processed breads are available and may be more recommended to patients with IBD. Further understanding of the role of FAs in IBD etiology may guide dietary recommendations.

## 1. Introduction

Inflammatory bowel disease (IBD), specifically Crohn’s disease (CD) and ulcerative colitis (UC), are chronic inflammatory conditions of the gastrointestinal tract. Over recent decades, the incidence and prevalence of IBD have increased, both in Western countries and newly industrialized countries [1,2,3]. One of the risk factors associated with this surge is the “Western diet” [4,5], characterized by a high intake of ultra-processed foods (UPFs), refined sugars, saturated fats, and food additives (FAs), such as preservatives and emulsifiers [6,7,8,9]. Notably, high emulsifier intake was previously documented in the habitual diet of patients with CD [10].

A central component of the Western diet is bread, accounting (together with cereals and pastries) for 2.2% of monthly food spending in Israel [11]. In the United States, bread is ranked amongst the most consumed grain products across all age groups [12]. Recent data from our group further support bread’s dietary relevance in patients with IBD: among 271 patients with newly diagnosed CD, the median intake of bread products was 1.85 servings/day (IQR: 0.71–3.51), with only few reports of no bread consumption at all. These findings emphasize the widespread intake of bread among patients with IBD [13].

Previous studies have linked a higher intake of ultra-processed breads—the largest contributors to overall UPF consumption—to an increased risk of CD. Specifically, individuals in the highest quartile of UPF intake had a significantly greater risk of CD, with ultra-processed breads showing one of the strongest associations [14]. Other findings suggest that ultra-processed breads are also associated with a higher probability of active disease among patients with IBD [15].

These studies relied on the NOVA classification system; NOVA is widely accepted as one of the few recognized food classifications systems [16]. Despite its prominence, NOVA may oversimplify the classification of certain foods. For example, most breads—except freshly made, unpackaged varieties—are classified as the 4th level ‘ultra-processed’, regardless of differences in FAs composition [17]. This broad categorization may obscure potential differences in the biological effects of different FAs.

Given how commonly bread is consumed, the frequent questions raised by patients with IBD, and earlier studies pointing to a possible link between industrial bread and IBD, we aimed to assess the availability of low-processed breads in Israel. This involved screening commercially available breads and categorizing them based on their composition and level of processing. Additionally, we sought to explore the existing literature on FAs found in industrial breads and their potential impact on inflammation and gut dysbiosis.

## 2. Methods

### 2.1. Breads Screening

This work was conducted as part of the IBDMED study (NCT05536544), which investigates the role of the Mediterranean diet in patients with IBD. Given that bread is a widely consumed staple food and included in the whole grains category of the Mediterranean diet, we examined the availability of low-processed bread options as preferable choices.

Commercially available breads were screened by IBD dietitians for their ingredient composition and FAs content, based on data retrieved from the online websites of two national chain stores during January–March 2023. The two chains are among the most prominent in their respective sectors and hold a big share of Israeli grocery markets, with significant national reach, multiple stores nationwide, and a broad product selection. One chain represents a large, mainstream supermarket with wide distribution across Israel, while the other is a health food store that caters to a more niche, health-conscious consumer base. Both chains operate online platforms with detailed product information, allowing for consistent and reliable ingredient extraction.

All bread products including buns and pita breads with a detailed product description and ingredient list were included in the analysis and coded. Data extracted included their brand names, weight, and prices in Israeli Shekels (ILS). The products were also divided into three groups according to the store they were available in—general/health food/both. Bread prices and weights were summarized using basic descriptive statistics, including means and standard deviations (SD±).

### 2.2. Ingredients Analysis

After mapping all ingredients, FAs were cross-referenced against the Food and Agriculture Organization of the United Nations (FAO)/World Health Organization (WHO) Codex of general standards for FAs (GSFA) [18] and against the food industry guidelines of the Ministry of Health in Israel [19]. We excluded flour and salt, which are part of the basic bread recipe, as well as nuts and seeds, which are unprocessed by nature.

Each of the FAs identified within the bread products was first assigned to its functional group (emulsifier/preservative/stabilizer/acidity regulator, etc.). In addition to Codex-defined FAs, our classification included certain technological ingredients—such as added gluten, palm oil, or added fibers—that are not formally categorized as FAs under the Codex but are widely used in industrial processing and are generally not used in home-baking. These ingredients are being referred to as FAs based on their relevance to food processing levels. Then, the different ingredients and FAs used in the breads were assigned into three categories. The green category included ingredients typically used in home-made breads such as yeast, sourdough, sugar, and olive or canola oil; the orange category included FAs and technological ingredients that extend beyond the basic bread recipe like malt, enzymes, and added fibers whose health implications are generally considered less concerning; the red category included FAs and technological ingredients used in the food industry, reflecting higher levels of processing like emulsifiers, preservatives, sweeteners, or added gluten, as detailed in Table 1 [20].

Each bread was then categorized into one of three categories according to the next rule—if it contained at least one ‘red’ ingredient, it was categorized as highly processed; if it contained no ‘red’ ingredients but at least one ‘orange’ ingredient, it was categorized as medium-processed; and if it did not contain ‘red’ or ‘orange’ FAs or ingredients, it was categorized as low-processed. The price for 100 g of bread was calculated from the total weight and price of each product. The Kruskal–Wallis test was used in order to compare differences across the three bread categories.

### 2.3. Literature Review

We conducted a literature review using PubMed to explore links between the identified FAs and IBD, inflammation, or gut dysbiosis. Key terms included “inflammation,” “gut microbiome,” “IBD,” and “dysbiosis,” combined with the FAs found in industrial breads. For each FA, we searched using both its E-number and common name, limiting results to English-language publications. To minimize bias, we prioritized peer-reviewed, recent studies and included both positive and negative findings. This ensured a comprehensive evaluation of potential associations or mechanisms linking these FAs to the targeted health outcomes. To ensure relevance to the field of IBD, we included studies with implications for gut inflammation, dysbiosis, or barrier dysfunction. Studies were excluded if they focused on non-human models with limited applicability to IBD, or investigated outcomes beyond the scope of gut inflammation. We excluded from the literature review ingredients from the green category as well as FAs that belonged to the red category where ingredient specificity was lacking, like ‘flavorings’ or ‘baking improver’.

## 3. Results

### 3.1. Most Breads Are Highly Processed and Contain Multiple FAs

We screened a total of 240 different bread products; of these, seven were excluded due to insufficient information or duplicate products listed under different names. Overall, 233 breads were included in the final analysis. Of these, 160 (68%) were available at the supermarket, 56 (25%) at the health food store, and 17 (7%) were available in both.

**Figure 1:**
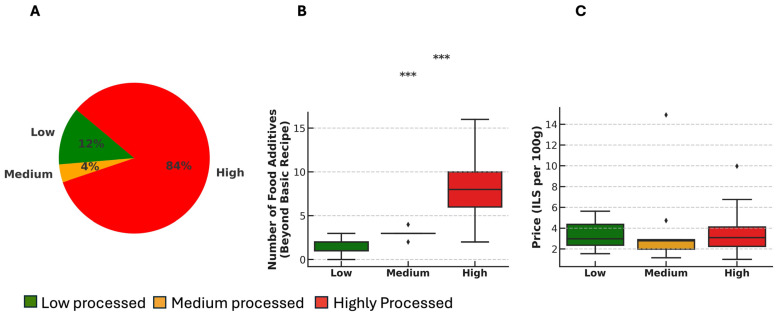
Food processing levels of industrial breads. (**A**) Levels of food processing. All screened breads were assigned into one of three categories: low processed (green), medium processed (orange), or highly processed (red). (**B**) Number of FAs in each category beyond the basic bread recipe, compared across processing levels using the Kruskal–Wallis test followed by Dunn’s post hoc analysis. (**C**) Bread prices per 100 g compared across processing levels using the Kruskal–Wallis test. *** *p* < 0.001.

The bread composition analysis showed that most breads—195 (84%)—were classified as highly processed, 9 (4%) as medium-processed, and 29 (12%) as low-processed (Figure 1A). Across these breads, we identified 37 different types of FAs and ingredients used in the bread industry in Israel, in addition to wheat flour, salt, nuts and seeds, which were excluded from this analysis since they form the basic bread recipe. While salt was not included in the FAs classification—given its role in the basic bread recipe—it was quantified separately due to its nutritional relevance, particularly its contribution to dietary sodium intake. Notably, it was present in 230 (99%) of breads.

The number of FAs and functional ingredients varied significantly across bread processing levels. Median FA counts in low-, medium-, and highly processed breads were 1 (IQR: 1–2), 3 (IQR: 3–4), and 8 (IQR: 6–10), respectively (*p* < 0.001) (Figure 1B). Notably, all highly processed breads contained more than one red ingredient, reflecting how breads within this category often include multiple FAs. The most common FA in the low-processed category was yeast, present in 199 (85%) of breads.

Bread prices were comparable across the three processing level groups. The median weight of a loaf of bread was 556 g (IQR: 530–631 g). When calculated per 100 g, the median prices for low-, medium-, and highly processed breads were 2.98, 2.78, and 3.09 ILS/100 g, respectively (*p* = 0.434) (Figure 1C).

### 3.2. Preservatives and Emulsifiers Are Dominant in Industrial Breads

**Figure 2:**
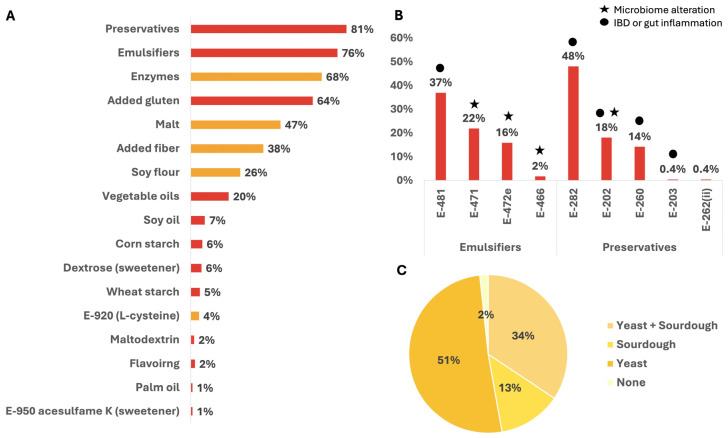
Prevalence of FAs and technological ingredients in industrial breads. (**A**) Commonly used FAs and technological ingredients. Prevalence rates of FAs and technological ingredients in industrial breads (n = 233). The colors represent the classification of each FA to a different processing level, medium processed (orange) or highly processed (red). FAs, food additives. (**B**) Prevalence of emulsifiers and preservatives in breads. The two groups of the most common additives were furthermore analyzed. A star or a circle signifies if it was linked to microbiome alterations or IBD or gut inflammation, respectively, in the literature review. E-481, Sodium stearoyl lactylate; E-471, Mono- and diglycerides of fatty acids; E-472e, DATEM; E-466, Sodium carboxy methyl cellulose (CMC); E-282, Calcium propionate; E-202, Potassium sorbate; E-260, Acetic acid; E-203, Calcium sorbate; E-262(ii), Sodium diacetate. (**C**) The prevalence of leavening agents. Yeast (n = 119), Yeast + Sourdough (n = 80), Sourdough (n = 30), None (n = 4).

Next, we analyzed the prevalence of different FAs and technological ingredients used. Most breads contained preservatives—89 (81%), and emulsifiers—178 (76%). Enzymes were found in 159 (68%) and added gluten in 148 (64%). The other commonly used FAs were malt in 109 (47%), added fiber in 88 (38%), soy flour in 60 (26%), and vegetable oils in 46 (20%) breads. Some of the FAs recognized were found in less than 10% of breads, like corn starch or wheat starch used in 14 (6%) and 11 (5%) breads, respectively (Figure 2A).

As preservatives and emulsifiers were the most prevalent FAs used, we further analyzed their different types (Figure 2B). A total of five preservatives were identified; of them, the most common was calcium propionate (E-282) found in 112 (48%) breads. Another two common preservatives were potassium sorbate (E-202) found in 42 (18%) breads and acetic acid (E-260) in 33 (14%) breads. The other two preservatives, calcium sorbate (E-203) and sodium diacetate [E-262(ii)], were rarely used, present only in one product each. A total of four emulsifiers were identified; the most used emulsifier was sodium stearoyl-2-lactylate (SSL, E-481) present in 86 (37%) breads, exclusively used in breads sold in supermarkets, with none detected in health food store breads. Alongside SSL (E-481), there were three other emulsifiers used; mono- and diglycerides of fatty acids (MDGs, E-471), previously reported as the largest contributor to emulsifier exposure in patients with IBD and healthy controls [21], were found in 22% of breads, while diacetyl tartaric acid esters of mono- and diglycerides (DATEM, E-472e) were present in 16% breads. Interestingly, carboxymethyl cellulose (CMC, E-466), extensively researched in IBD and related to the induction of gut dysbiosis and reduced α-diversity [10,22,23], was found in only 2% of industrial breads (Table 2).

We next compared the use of the two most common leavening agents, both considered low-processed, yeast and sourdough. Most breads utilized yeast or a combination of yeast and sourdough as leavening agents. Breads leavened using yeast were more prominent—119 (51%), followed by products which used both yeast and sourdough—80 (34%). A small number of 30 (13%) breads used only sourdough, and 4 (2%) breads did not use any leavening agent (Figure 2C).

### 3.3. Prevalent FAs May Have Implications on Inflammation or Gut Microbiome

Finally, to assess the potential implications of FAs used in bread industry, we conducted a literature review to explore the association between FAs, intestinal inflammation and gut microbiome. The review included 53 articles, all experimental studies using different models. The largest group comprised animal studies (n = 29), including various mouse models (DSS-induced colitis, IL-10 knockout, Rag1 knockout, and Casp8ΔIEC mice). Human studies or human samples (n = 14) included clinical trials with IBD patients, healthy subjects, and studies using human microbiota in specialized systems. In vitro studies (n = 11) utilized various models including cell cultures (Caco-2 cells, RAW 264.7 macrophages) and fecal microbiota cultures. These experimental studies examined how FAs affect inflammation and gut microbiome, while others investigated mechanisms by which specific FAs influence intestinal integrity and immune function. The literature review revealed that 19 FAs were associated with negative effects on inflammation or the gut microbiome. However, a closer examination showed that only 14 of these had consistently negative findings, while the remaining 5 demonstrated mixed outcomes. Ten ingredients showed potentially beneficial effects.

Preservatives, which were the most prominent FAs, like calcium propionate (E-282) and potassium sorbate (E-202) prevalent in 48% and 18% of products, respectively, exhibit immunological effects, including increased serum IgG and inflammatory cytokines [29]. Emulsifiers, including SSL (E-481), MDGs (E-471), and DATEM (E-472e), have been linked to alterations in gut microbiome diversity and composition, as well as increases in circulating lipopolysaccharides (LPS) and pro-inflammatory cytokines (e.g., IL-1β, IL-6, and TNF-α) [23,24,25]. Additionally, CMC (E-466), though present in only 2% of the products screened, demonstrates a potent effect on microbial gene expression, bacterial adherence, and inflammatory responses in various models [10,22,23,26,36]. These FAs contribute to an altered gut environment, which may exacerbate inflammatory conditions.

In addition to preservatives and emulsifiers, other FAs have shown similar effects. Palm oil, for example, has been reported to disrupt tight junction proteins and increase LPS translocation in Caco-2 cells, while also increasing flare risk in patients with UC in remission [37,38]. Animal studies have further demonstrated that palm oil can shift the microbiome toward increased *Proteobacteria* with decreased *Firmicutes* in the small intestine [39,40]. Some thickeners like maltodextrin increased biofilm formation of adherent-invasive *E. coli* and enhanced IL-1β production. Artificial sweeteners, like acesulfame potassium (E-950), have been linked to increased pro-inflammatory markers and microbial dysbiosis [41,42].

On the other hand, some FAs exhibit mixed effects. Guar gum (E-412), for instance, has demonstrated varied outcomes across different models—it reduced colonic inflammation and increased beneficial short-chain fatty acids in colitis models, yet also modified intestinal T cell responses in an immunosuppressed model and increased specific bacterial populations including *Clostridium* groups and *Bacteroides fragilis* [43,44]. In contrast, components of soy, including lunasin, phosphatidylcholine, and phytosterols, may support remission in IBD patients by promoting apoptosis via the NF-κB pathway, improving clinical responses and reducing colitis symptoms. Phytosterols, specifically, have been shown to inhibit the NF-κB pathway in intestinal epithelial cells and experimental murine colitis models. Additionally, phosphatidylcholine has demonstrated efficacy in increasing histological remission rates in patients with UC refractory to mesalazine [45,46,47,48]. Other compounds, such as citric acid (E-330) and ascorbic acid (vitamin C, E-300), provide potential benefits by promoting beneficial gut bacteria and exhibiting anti-inflammatory properties, respectively [49,50]. Lactic acid has been found to delay LPS-induced monocyte activation and inhibit TNF-α secretion, partially through interference with key signaling pathways, suggesting a role in modulating inflammatory responses [51]. These findings highlight the complex and often opposing effects of different FAs on gut health and inflammation, suggesting careful consideration is needed when evaluating their use, particularly in the context of IBD (Supplementary Materials, Table S1).

## 4. Discussion

Here, we provide an assessment of the composition of industrial breads available in Israel, alongside a review of the potential implications of their ingredients in IBD. Among the 233 breads analyzed, 84% were classified as highly processed, with an average of eight FAs, including FAs associated with inflammation and gut dysbiosis.

Given the large quantities of bread consumed, the rise in mass production has led to a shift away from traditional baking methods toward breads that more closely resemble the UPFs characteristic of the Western diet [9,52]. These changes raise concerns about potential long-term health impacts. Research indicates that the Western diet and high consumption of UPFs can induce gut dysbiosis and promote inflammation, contributing to the development of IBD [8,53,54,55]. Importantly, previous epidemiological observations have linked the consumption of highly processed bread with an increased risk of IBD, specifically CD development, disease relapse, and IBD-related complications [14,15,56].

The characterization of the screened industrial breads revealed notable differences in their ingredient lists compared to traditional breads. The industrial breads analyzed were primarily highly processed, with extensive use of FAs. FAs are often used in bread to improve shelf life and texture, yet they have been linked to negative health outcomes, such as gut microbiome dysbiosis and chronic inflammation [57,58,59]. Although we also identified low-processed breads in grocery stores/supermarkets, their availability was considerably lower than that of the highly processed ones.

The most common FAs used in industrial breads were preservatives and emulsifiers, many of which are primarily synthetic, such as SSL (E-481) and CMC (E-466). While some, like propionate, acetate, and mono- and diglycerides (MDGs, E-471), resemble naturally occurring metabolites, they may exert different effects. For example, orally ingested propionate has shown distinct metabolic impacts compared to microbiota-derived propionate and does not replicate its beneficial effects [60,61].

The literature review revealed that FAs found in most breads, particularly preservatives and emulsifiers, have been previously associated with inflammation and microbiome alterations. This was reflected by increased levels of LPS, IL-1β, IL-6, and TNF-α in serum, as well as increased intestinal epithelial barrier permeability [22,29,35,62]. Multiple studies have shown impact on the microbiome, including changes in bacterial load and composition, such as a decrease in members of *Akkermansia*, *Bifidobacterium*, *Lactobacillus*, and *Lupinus luteus* [23,24,63]. SSL, the most prominent emulsifier, found only in breads sold in the supermarket, was linked to microbiome alterations in vitro, including reduction in the relative abundance of *Clostridiaceae*, *Lachnospiraceae*, and *Ruminococcaceae* and increase in LPS [24]. Of note, calcium propionate (E-282) and potassium sorbate (E-202), used as preservatives, previously showed an increase in cytokine production such as TNF-α [29]. Interestingly, acetic acid (E-260), which was also commonly used in breads, showed mixed effects. While it has been used to induce colitis and oral inflammation in animal models [33], high concentrations of acetate demonstrated protective effects in a patient-derived human epithelial cell culture model, including improved epithelial resistance and reduced expression of IL8 and TNFα [34] (Table 2).

In addition, leavening agents may also affect inflammation. Dietary yeast was previously shown to alter T cell responses in ASCA-positive CD patients and increase IgA levels [64,65]. In Casp8^ΔIEC^ mice, yeast-leavened bread exacerbated colitis [66]. Conversely, sourdough bread has been associated with increased production of short-chain fatty acids [67]. As yeast is commonly used in homemade bread, it was initially included in the green category. However, considering the potential adverse effect of yeast on inflammation, sourdough might be a preferable leavening agent in a subset of patients with IBD.

Added gluten and dietary fiber are common technological ingredients present in 68% and 38% of breads, respectively. Both FAs have complex effects on gut health and inflammation. Current evidence does not support specific recommendations to restrict gluten in IBD management; however, some therapeutic diets, such as the Crohn’s Disease Exclusion Diet (CDED), CD-Treat, and The Specific Carbohydrate Diet (SCD), incorporate gluten avoidance as part of their protocol [68,69,70,71]. Gluten increases intestinal permeability via MyD88-dependent zonulin upregulation and reduces microbial α-diversity, particularly Firmicutes, in gluten-sensitive models [72,73,74]. In contrast, dietary fibers generally support gut health and IBD management, and their effects vary by subtype, solubility, and fermentation profile. In dysbiotic conditions, the inappropriate fermentation of fibers may exacerbate inflammation, underscoring the need for targeted research on these dietary components [75,76].

A noteworthy finding from our analysis is that salt was present in 230 (99%) of the breads examined, highlighting its widespread use in industrial bread manufacturing. Additionally, vitamin C, considered an antioxidant, was found in 137 (59%) of the breads, while no bread samples contained artificial food colorings. These components contribute to the overall nutritional and FA profiles of the breads and warrant further consideration in relation to health outcomes.

Although the specific FAs identified in breads are regulated in terms of permitted levels by national authorities such as the Ministry of Health [19], the actual exposure from daily consumption is not routinely monitored or well quantified. Notably, patients with IBD in Israel consume bread daily, and it may serve as a constant contributor to FAs exposure. While our study did not assess intake levels of FAs directly, their cumulative effects on gut microbiome and mucosal immunity remain a concern for both the prevention and maintenance of remission in IBD [13,15,21,56,71,77].

The majority of breads were highly processed, yet low-processed bread options were identified at both the supermarket and health food stores, though their selection was limited. In the low-processed category, we included products with minimal ingredients listed, considering them the closest alternatives to homemade bread with only 12% of the products meeting this criterion. The scarcity of low-processed breads highlights the need for future initiatives, such as encouraging the food industry to develop improved alternatives. Notably, we identified variability in the reported effects of different ingredients and FAs. For example, phosphatidylcholine, a component in soy flour, which is used as an emulsifier, was previously shown to improve clinical and histological scores in patients with UC [46]. This may serve as a better alternative to other emulsifiers like MDGs (E-471), which were shown to increase pro-inflammatory cytokines in animal models [25]. As presently the consumption of UPFs is practically inevitable, the inclusion of options with “safer” FAs is warranted.

In contrast to the Western diet, the Mediterranean diet emphasizes whole grains, fresh fruits, and vegetables, legumes, and healthy fats, particularly from olive oil, while limiting the consumption of UPFs [7,78]. It is characterized by minimally processed, nutrient-dense foods that support overall health and benefit the microbiome [79,80,81]. The Mediterranean diet is one of the recommended approaches for patients with IBD management globally and in Israel specifically [13,82,83,84,85,86,87,88]. As bread is a common and accessible source of whole grains in the Mediterranean diet, selecting minimally processed bread should be emphasized in nutritional recommendations and patient education regarding grocery shopping habits [81,82,89,90].

Notably, low-processed bread, which is usually perceived as having health benefits, is often priced competitively, counterintuitive to the prevalent assumption that healthier food options are generally more expensive.

The strength of our study lies in its comprehensive screening of all ingredients used in industrial breads in Israel. Study researchers, including dietitians trained in IBD, meticulously registered and categorized FAs into three distinct categories, noting their reported relation to IBD based on a literature review. This process and detailed categorization address the limitations of the NOVA classification system, which aggregates all FAs into the 4th ‘ultra-processed’ category without distinguishing between different ingredients. Although UPFs are prevalent in the Western diet, no universally accepted classification system exists. Frameworks like NOVA categorize foods based on their extent of processing but often fail to account for the specific health impacts of individual FAs [17,91]. Our study suggests a more “IBD-relevant” categorization should be considered, based on evidence from animal and human studies of IBD, and highlights the need for a more nuanced analysis of these FAs.

We acknowledge that our study has several limitations. First, bread categorization was based on broad nutritional criteria, and the associations between FAs, inflammation, and gut microbiome were based on basic science studies rather than clinical trials in patients with IBD. In the absence of screening methodologies to assess the immunological safety of FAs, translating basic science into nutritional recommendations may serve as a bridge for tailored and population-targeted recommendations. Second, our study focused on industrial breads in Israel, potentially reflecting a local phenomenon. Yet, our results align with findings from other Western populations. A recent Australian analysis categorized products from a packaged-food database and found that only 31.5% of 238 breads were free from emulsifiers or thickeners. Interestingly, the emulsifiers used in Australian breads were similar to those used in Israel, such as MDGs (E-471), SSL (E-481), and DATEM (E-472e), underscoring common practices in the bread industry across Western societies [77]. Finally, while we mapped the FAs used in industrial bread, we did not assess the actual quantities to which individuals are exposed. For instance, acetic acid (E-260) is regulated in bread, with levels lower than a tablespoon of vinegar per loaf. While this suggests minimal exposure per serving, the actual intake may vary depending on individual consumption patterns. Moreover, we cannot quantify the cumulative effect of daily bread consumption over time. Given bread’s ubiquity in diets and data from a recent cohort of patients with newly diagnosed CD, showing that its consumption among patients with IBD is prominent, further assessment is needed to evaluate the long-term impact of such exposures. Additionally, our analysis relied on product label declarations, which may not capture all FAs present in trace amounts below regulatory thresholds. As a result, the presence of certain FAs may be underreported, and our dataset likely reflects a conservative estimate of FAs exposure. Still, from a clinical and consumer perspective, these limitations are consistent with the level of information available to health professionals and the public in real-life decision making.

## 5. Conclusions

This study provides an analysis of the composition of industrial breads in Israel, revealing that the majority are highly processed and contain multiple FAs previously associated with gut microbiome alterations and inflammatory responses. Our findings emphasize the importance of considering dietary factors such as industrial bread composition in the dietary management of patients with IBD. While further research is needed to establish definitive conclusions, particularly regarding the cumulative effects of FAs consumption, we suggest that dietary choices should prioritize low-processed foods. Additionally collaborative efforts with the food industry to explore the use of FAs with no previously reported immunological effects could be a step toward improving the nutritional quality of industrial breads. The data collected and tabulated may be used by health care practitioners and patients alike for making dietary choices. As findings from longitudinal studies incorporating comprehensive dietary assessments and clinical outcomes are reported, this categorization can be updated. Such information may be essential to clarify the potential role of industrial bread consumption in the development and management of IBD.

## Figures and Tables

**Table 1:** The three different categories of FAs and ingredients used in bread products. The green category considered as ‘low processed’ includes ingredients typically found in homemade breads, the orange category—‘medium processed’—includes ingredients that extend beyond the basic bread recipe, and the red category includes both officially defined FAs and technological ingredients that contribute to the overall processing level. * Yeast is commonly used in homemade bread; therefore, it was categorized as “green”.

Low Processed	Medium Processed	Highly Processed
Baking soda	Added fiber	Acidity regulators
Canola oil	Enzymes	Added gluten
Olive oil	L-cysteine	Anticaking agents
Sourdough	Malt	Baking improver
Sugar	Soy flour	Emulsifiers
Vitamin C		Flavorings
Yeast *		Palm oil, soy oil, or unspecified vegetable oil
		Preservatives
		Stabilizers
		Sweeteners: acesulfame K, maltodextrin, dextrose
		Wheat starch or corn starch

**Table 2:** Emulsifiers and preservatives present in industrial breads, their prevalence, and possible effects on microbiome or inflammation. ↓—decrease, ↑—increase.

Group	Food Additive	E-Number	Prevalence (in Screened Breads) (%)	Model	Effect on Microbiome	Effect on Inflammation	Ref
Emulsifiers	SSL	E-481	37%	In vitro—fecal microbiota	↓ *Clostridiaceae*, *Lachnospiraceae*, *Ruminococcaceae* ↓ Butyrate ↑ *Bacteroidaceae*, *Enterobacteriaceae* ↑ Propionate ↑ LPS and flagellin		[24]
Emulsifiers	MDGs	E-471	22%	Murine model—mice	Changes β-diversity and microbial composition ↓ *Akkermansia*, *Bifidobacterium*, *Lactobacillus*, *Lupinus luteus* ↑ *Bacteroides acidifaciens*, *E. coli*	↑ LPS, IL-1β, IL-6, and TNF-α levels in serum	[25]
Emulsifiers	DATEM	E-472e	16%	Human microbiota—MBRAs	↓ Bacterial density ↓ *Lactobacillales* members, including *Streptococcus* genus ↓ *Faecalibacterium*		[23]
Emulsifiers	CMC	E-466	2%	M-SHIME, murine model—mice	↑ Bioactive flagellin-related gene expressions ↑ IL-6 expression	↑ Intestinal inflammation	[22]
Emulsifiers	CMC	E-466	2%	MBRAs	↓ *Lactobacillales* members, *Streptococcus* genus		[23]
Emulsifiers	CMC	E-466	2%	Murine model—mice	↑ Bacterial adherence ↑ Bacterial overgrowth		[26]
Emulsifiers	CMC	E-466	2%	Murine model—mice	↑ *E. coli* ability to adhere and invade IEC ↑ Expression of virulence factors		[27]
Emulsifiers	CMC	E-466	2%	Murine model—mice	↑ Bioactive fecal LPS and flagellin	↑ Shortened colons ↑ Splenomegaly	[10,28]
Preservatives	Calcium propionate	E-282	48%	Murine model—rats		**In serum:** ↓ IgG and IgM ↑ IL-4 expression **mRNA expression:** ↑ TNF-α expression	[29]
Preservatives	Potassium sorbate	E-202	18%	Murine model—rats		**In serum:** ↓ IgG and IgM ↑ IL-4 expression **mRNA expression:** ↑ TNF-α expression and IFNγ	[29]
Preservatives	Potassium sorbate	E-202	18%	Murine model—mice	↓ α-diversity ↑ *Parabacteroides* and *Adlercreutzia*		[30]
Preservatives	Potassium sorbate	E-202	18%	In vitro—fecal microbiota	↓ *E. Coli*		[31]
Preservatives	Potassium sorbate	E-202	18%	Murine model—mice	↓ *Lachnospiraceae* ↓ Isobutyric acid production	↑ IL-1β levels in serum ↑ Inflammatory cell infiltration in the liver	[32]
Preservatives, Acidity regulators	Acetic acid	E-260	14%	Murine model—rats		↑ Colitis	[33]
Preservatives, Acidity regulators	Acetic acid	E-260	14%	Organoid-derived colonic epithelial monolayer culture		↑ Improved epithelial resistance ↑ Regulation of HIF1α, MUC2, and MKI67 ↓ Expression of: IL8, TNFα, CLDN1	[34]
Preservatives, Acidity regulators	Sodium hydrogen acetate	E-262(ii)	0.40%	Murine model—rats		↓ IgG and IgM levels ↓ PPAR-α, PPAR-γ expression ↑ TNF-α expression	[35]

SSL, stearoyl-2-lactylate; LPS, lipopolysaccharides; MDGs, mono- and diglycerides of fatty acids; *E. coli*, *Escherichia coli*; DATEM, diacetyl tartaric acid esters of mono- and diglycerides of fatty acids; MBRAs, Mini Bio Reactor Arrays; CMC, carboxymethyl cellulose; M-SHIME, Simulator of the Human Intestinal Microbial Ecosystem; IEC, intestinal epithelial cells.

## Data Availability

The original contributions presented in the study are included in the article/Supplementary Materials, further inquiries can be directed to the corresponding author.

## Supplementary Materials

The following supporting information can be downloaded at: https://www.mdpi.com/article/10.3390/nu17132120/s1, Table S1: FAs present in industrial breads, organized from the most to the least prevalent ingredient and their possible effects on microbiome or inflammation. ↓—decrease, ↑—increase. References [91,92,93,94,95,96,97,98,99,100,101,102,103,104,105,106,107,108] are cited in the Supplementary Materials.
