# Supplementary material for: Industrial Bread Composition: Potential Implications for Patients with Inflammatory Bowel Disease
Source: Nutrients. 2025 Jun 26;17(13):2120. doi: 10.3390/nu17132120 (PMC12252189; doi:10.3390/nu17132120)
Supplement: Supplementary file 1 [file nutrients-17-02120-s001.zip › nutrients-3689230-supplementary.pdf]

Supplementary materials

Table S1 FAs present in industrial breads, organized from the most to the least prevalent ingredient and their possible effects on microbiome or inflammation. ↓ - decrease, ↑ - increase.

Group	Food additive	E-number	Prevalence (in screened breads) (%)	Model	Effect on microbiome	Effect on inflammation	Ref
Raising agents	Yeast	NA	85%	Murine model - rats		↑ IL-6 and TNF-α levels in serum	[1]
Raising agents	Yeast	NA	85%	Human: IBD vs. healthy subjects		*Saccharomyces cerevisiae*:<br>↑ reactivity of CD45RA− memory T cells in CD<br>↑ CD4+ T cell responses<br>↑ Cytotoxic TH1 cells<br>Altered T cells in patients with CD who are ASCA+	[2]
Raising agents	Yeast	NA	85%	Human: IBD vs. healthy subjects		Yeast cell wall mannan:<br>↑ IgA, IgM and IgG levels in CD and UC	[3]
Raising agents	Yeast	NA	85%	$Casp8^{\Delta IEC}$ mice		↑ Colitis<br>↑ Mortality	[4]
Flavor improver (texture, structure, shelf life)	Enzymes (amylase, lipase, and glucose oxidase)	NA	68%	Piglets	Glucose oxidase - ↓ *Fusobacteriota*, *Fusobacteriia*, *Fusobacteriales*, *Fusobacteriaceae*	Glucose oxidase - in the duodenum<br>↓ IL-8 and ileal IL-6 expression<br>↓ TNF-α expression	[5]
Flavor improver	Enzymes	NA	68%	Human		↓ IL-6 concentrations in serum	[6]
Improves structure, texture	Gluten	NA	64%	Intestinal mucosal membrane		↑ MyD88-dependent zonulin<br>↑ Intestinal permeability	[7]
Improves structure, texture	Gluten	NA	64%	Gluten-sensitive juvenile macaque model	↓ Gut microbial α-diversity – mainly *Firmicutes* phylum		[8]
Antioxidants	Ascorbic acid (vitamin C)	E-300	59%	Mice with DSS induced UC		↓ NF-κB, COX-2 and iNOS in the colon<br>↓ TNF-α, IL-1β, IL-6 and IL-17 in the colon	[9]

Flavor improver (texture, structure, shelf life)	Malt	NA	47%	Human		↑ Antisecretory factor (AF)<br><br>↓ Intestinal inflammation	[10]
Emulsifiers	Soy flour	NA	26%	RAW 264.7 macrophages		Lunasin (present in soy flour) -<br>↑ Proapoptotic genes in an NF-κB–dependent manner	[11]
Emulsifiers	Soy flour	NA	26%	Mesalazine-refractory patients		Phosphatidylcholine (present in soy flour) -<br>↑ Clinical response<br>↑ Histological remission<br>↑ Remission rate	[12]
Emulsifiers	Soy flour	NA	26%	IEC & Murine colitis models		Phytosterols (present in soy flour) -<br>↓ Colitis symptoms<br>↓ NF-κB pathway<br>↓ Binding of LPS to TLR4 in peritoneal macrophages	[13]
Emulsifiers	Soy flour	NA	26%	Rag1 knockout mice		Phytosterols (present in soy flour) -<br>↑ Regulatory T cells in colon tissue<br><br>↓ CD3+ T-cell positive staining in mice	[14]
Acidity regulators, Antioxidants	Citric acid	E-330	11%	Murine model - mice	↑ *Bifidobacterium* & *Lactobacillus*	↑ Proliferation of epithelial cells<br>↑ Integrity of tight junctions<br>↑ Small intestinal villus–crypt ratio	[15]
Emulsifier, Natural flavors enhancer	Soy oil	NA	7%	Murine model - mice	↑ Gut dysbiosis<br>↑ Endogenous adherent invasive E.coli	↑ Immune dysfunction<br>↑ Intestinal epithelial barrier permeability<br><br>↓ Balance of Hepatocyte Nuclear Factor 4α (HNF4α) isoforms	[16]
Food colorant Anti-caking agents Stabilizers	Calcium carbonate	E-170	7%	Human rectal mucosa		↓ COX-2 expression in whole crypts<br><br>↑ 15-HPGD expression	[17]
Improves yeast fermentation, color, flavor, texture and shelf life	Dextrose	NA	6%	Murine model - rats	Alterations in microbial community	↑ IgA secretion in the caecum<br>↑ Propionic acid<br>↑ Ileal IL-1-positive cells<br>↑ Caecal TGF- -positive cells<br><br>↓ Caecal pH<br>↓ Acetic acid	[18]

						↓ Branched SCFA<br>↓ Villus height and depth	
Thickener (texture, structure)	Corn starch	NA	6%	Human	↑ *Firmicutes*<br>↑ Butyrate and propionate<br><br>↓ *E. Coli*		[19]
Thickener, stabilizer, binding agent	Wheat starch	NA	5%	Murine model - mice	↑ *Parabacteroides*, *Faecalibaculum*, and *Muribaculum*<br><br>↓ *Colidextribacter*	↓ IL-1β, IL-6 in serum	[20]
Thickeners, stabilizers	Guar gum	E-412	4%	Murine colitis induced	↑ *Clostridium coccoides* group, *Clostridium leptum*, *Bacteroides fragilis*	↓ Colonic damage<br>↓ MPO activity<br>↓ TNF-α and mRNA expression in colonic mucosa<br><br>↑ Acetic acid, propionic acid, butyric acid in cecum	[21]
Thickeners, stabilizers	Guar gum	E-412	4%	cyclophosphamide (CTX)-induced immunosuppressed		↑ Immune suppression in intestinal mucosal T cells	[22]
Thickeners, stabilizers	Guar gum	E-412	4%	DSS-induced colitis mice	↑ *Akkermansia muciniphila* and *Oscillospira*	↓ Inflammatory cytokine levels<br><br>↑ Short-chain fatty acids (SCFAs) production	[23]
Flour treatment agents	L-cysteine	E-920	4%	Mice with DSS induced UC		↓ TNF-alpha, IL-6, IL-12p40, IL-1beta<br>↓ Intestinal permeability<br>↓ Local chemokine expression and neutrophil influx<br><br>Improved colon histology	[24]
Flour treatment agents	L-cysteine	E-920	4%	IL-10 KO mice	↑ *Enterobacteriaceae, Klebsiella*		[25]
Thickeners, stabilizers	Xanthan gum	E-415	3%	MBRAs	↑LPS levels in microbiota		[26]

					↑ Bioactive levels of flagellin		
Thickeners, stabilizers	Xanthan gum	E-415	3%	DSS-induced colitis mice	↑ *Ruminococcus gnavus*		[23]
Thickener, filler	Maltodextrin	NA	2%	MBRAs	↑ LPS levels in microbiota		[26]
Thickener, filler	Maltodextrin	NA	2%	Murine model - mice	↓ Response to *Salmonella* infection		[27]
Thickener, filler	Maltodextrin	NA	2%	Murine model - mice		↑ IL-1β and Lcn-2<br>↑ Endoplasmic reticulum (ER) stress<br><br>↓ Muc-2 staining<br>↓ Glycosylated (mature) Muc-2	[28]
Thickener, filler	Maltodextrin	NA	2%	Gastrointestinal tissue	↑ Biofilm formation AIEC strain LF82		[29]
Thickener, filler	Maltodextrin	NA	2%	Murine model - mice		Resistant Maltodextrin:<br>↑ Intestinal barrier damage brought by DSS<br><br>↓ IFN-γ and TNF-α production induced by DSS	[30]
Acidity regulators	Lactic acid	E-270	1%	Human monocytes		↓ LPS-induced monocyte activation<br>↓ TNF-α secretion and glycolysis	[31]
Acidity regulators Raising agents	Sodium bicarbonate (baking soda)	E-500ii	1%	Human and murine model - rats		↑ Anti-inflammatory response	[32]
Sweeteners	Acesulfame potassium (K)	E-950	1%	Human	↑ *Proteobacteria*, specifically *Enterobacteriaceae*<br>↑ *Bacteroides* genus		[33]
Sweeteners	Acesulfame potassium (K)	E-950	1%	Murine model - mice	↑ Dysbiosis	↑ Proinflammatory cytokines<br>↑ Small intestinal injury<br>↑ Intestinal permeability<br>↑ Migration of lymphocytes to intestinal micro vessels	[34]
Emulsifiers, Flavor improver (texture,	Palm oil	NA	1%	Caco-2 cells		↑ Chylomicron secretion<br>↑ LPS translocation	[35]

structure, shelf life)							
Emulsifiers, Flavor improver	Palm oil	NA	1%	UC patients in remission		↑ Risk of flare	[36]
Emulsifiers, Flavor improver	Palm oil	NA	1%	Pigs	↑ *Proteobacteria*<br><br>↓ *Firmicutes* (small intestine)		[37]
Emulsifiers, Flavor improver	Palm oil	NA	1%	Caco-2 cells		Effects the disassembly of the tight junction and adherent proteins<br><br>↑ FD-4 permeability across the epithelial barrier	[38]

References

1. Dangarembizi R, Erlwanger KH, Rummel C, Roth J, Madziva MT, Harden LM. Brewer's yeast is a potent inducer of fever, sickness behavior and inflammation within the brain. *Brain Behav Immun*. 2018;68:211-223. doi:10.1016/j.bbi.2017.10.019
2. Martini GR, Tikhonova E, Rosati E, et al. Selection of cross-reactive T cells by commensal and food-derived yeasts drives cytotoxic TH1 cell responses in Crohn's disease. *Nat Med*. 2023;29(10):2602-2614. doi:10.1038/s41591-023-02556-5
3. Lindberg E, Magnusson KE, Tysk C, Jarnerot G. Antibody (IgG, IgA, and IgM) to baker's yeast (Saccharomyces cerevisiae), yeast mannan, gliadin, ovalbumin and betalactoglobulin in monozygotic twins with inflammatory bowel disease. *Gut*. 1992;33(7):909-913. doi:10.1136/gut.33.7.909
4. Zimmermann J, De Fazio L, Kaden-Volynets V, Hitzmann B, Bischoff SC. Consumption of Yeast-Fermented Wheat and Rye Breads Increases Colitis and Mortality in a Mouse Model of Colitis. *Dig Dis Sci*. 2022;67(9):4422-4433. doi:10.1007/s10620-022-07462-3
5. Wang W, Xie R, Cao Q, et al. Effects of glucose oxidase on growth performance, clinical symptoms, serum parameters, and intestinal health in piglets challenged by enterotoxigenic Escherichia coli. *Front Microbiol*. 2022;13. doi:10.3389/fmicb.2022.994151
6. Paradis ME, Couture P, Gigleux I, Marin J, Vohl MC, Lamarche B. Impact of systemic enzyme supplementation on low-grade inflammation in humans. *PharmaNutrition*. 2015;3(3):83-88. doi:10.1016/j.phanu.2015.04.004
7. Lammers KM, Lu R, Brownley J, et al. Gliadin Induces an Increase in Intestinal Permeability and Zonulin Release by Binding to the Chemokine Receptor CXCR3. *Gastroenterology*. 2008;135(1):194-204.e3. doi:10.1053/j.gastro.2008.03.023
8. Mohan M, Chow CE, Ryan C, et al. Dietary Gluten-Induced Gut Dysbiosis Is Accompanied by Selective Upregulation of microRNAs with Intestinal Tight Junction and Bacteria-Binding Motifs in Rhesus Macaque Model of Celiac Disease. *Nutrients*. 2016;8(11):684. doi:10.3390/nu8110684
9. Yan H, Wang H, Zhang X, Li X, Yu J. Ascorbic acid ameliorates oxidative stress and inflammation in dextran sulfate sodium-induced ulcerative colitis in mice. *Int J Clin Exp Med*. 2015;8(11):20245-20253. http://www.ncbi.nlm.nih.gov/pubmed/26884937
10. Bjorck S. Food induced stimulation of the antisecretory factor can improve symptoms in human inflammatory bowel disease: a study of a concept. *Gut*. 2000;46(6):824-829. doi:10.1136/gut.46.6.824
11. Dia VP, Gonzalez de Mejia E. Differential gene expression of RAW 264.7 macrophages in response to the RGD peptide lunasin with and without lipopolysaccharide stimulation. *Peptides (NY)*. 2011;32(10):1979-1988. doi:10.1016/j.peptides.2011.09.009
12. Karner M, Kocjan A, Stein J, et al. First Multicenter Study of Modified Release Phosphatidylcholine "LT-02" in Ulcerative Colitis: A Randomized, Placebo-Controlled Trial in Mesalazine-Refractory Courses. *American Journal of Gastroenterology*. 2014;109(7):1041-1051. doi:10.1038/ajg.2014.104
13. Cheon JH, Kim JS, Kim JM, Kim N, Jung HC, Song IS. Plant sterol guggulsterone inhibits nuclear factor-κB signaling in intestinal epithelial cells by blocking IκB kinase and ameliorates acute murine colitis. *Inflamm Bowel Dis*. 2006;12(12):1152-1161. doi:10.1097/01.mib.0000235830.94057.c6

14. Te Velde A, Brüll F, Heinsbroek S, et al. Effects of Dietary Plant Sterols and Stanol Esters with Low- and High-Fat Diets in Chronic and Acute Models for Experimental Colitis. *Nutrients*. 2015;7(10):8518-8531. doi:10.3390/nu7105412
15. Hu P, Yuan M, Guo B, et al. Citric Acid Promotes Immune Function by Modulating the Intestinal Barrier. *Int J Mol Sci*. 2024;25(2):1239. doi:10.3390/ijms25021239
16. Deol P, Ruegger P, Logan GD, et al. Diet high in linoleic acid dysregulates the intestinal endocannabinoid system and increases susceptibility to colitis in Mice. *Gut Microbes*. 2023;15(1). doi:10.1080/19490976.2023.2229945
17. Gibbs DC, Fedirko V, Baron JA, et al. Inflammation Modulation by Vitamin D and Calcium in the Morphologically Normal Colorectal Mucosa of Patients with Colorectal Adenoma in a Clinical Trial. *Cancer Prev Res (Phila)*. 2021;14(1):65-76. doi:10.1158/1940-6207.CAPR-20-0140
18. Peuranen S, Tiihonen K, Apajalahti J, Kettunen A, Saarinen M, Rautonen N. Combination of polydextrose and lactitol affects microbial ecosystem and immune responses in rat gastrointestinal tract. *Br J Nutr*. 2004;91(6):905-914. doi:10.1079/BJN20041114
19. Gerasimidis K, Nichols B, McGowan M, et al. The Effects of Commonly Consumed Dietary Fibres on the Gut Microbiome and Its Fibre Fermentative Capacity in Adults with Inflammatory Bowel Disease in Remission. *Nutrients*. 2022;14(5):1053. doi:10.3390/nu14051053
20. Chen X, Hou Y, Liao A, et al. Integrated Analysis of Gut Microbiome and Adipose Transcriptome Reveals Beneficial Effects of Resistant Dextrin from Wheat Starch on Insulin Resistance in Kunming Mice. *Biomolecules*. 2024;14(2):186. doi:10.3390/biom14020186
21. Takagi T, Naito Y, Higashimura Y, et al. Partially hydrolysed guar gum ameliorates murine intestinal inflammation in association with modulating luminal microbiota and SCFA. *British Journal of Nutrition*. 2016;116(7):1199-1205. doi:10.1017/S0007114516003068
22. Wu X, Huang X, Ma W, et al. Bioactive polysaccharides promote gut immunity via different ways. *Food Funct*. 2023;14(3):1387-1400. doi:10.1039/D2FO03181G
23. Zhang S, Sun Y, Nie Q, et al. Effects of four food hydrocolloids on colitis and their regulatory effect on gut microbiota. *Carbohydr Polym*. 2024;323:121368. doi:10.1016/j.carbpol.2023.121368
24. Kim CJ, Kovacs-Nolan J, Yang C, Archbold T, Fan MZ, Mine Y. L-cysteine supplementation attenuates local inflammation and restores gut homeostasis in a porcine model of colitis. *Biochimica et Biophysica Acta (BBA) - General Subjects*. 2009;1790(10):1161-1169. doi:10.1016/j.bbagen.2009.05.018
25. Richie TG, Heeren L, Kamke A, et al. Limitation of amino acid availability by bacterial populations during enhanced colitis in IBD mouse model. Methe B, ed. *mSystems*. 2023;8(6). doi:10.1128/msystems.00703-23
26. Naimi S, Viennois E, Gewirtz AT, Chassaing B. Direct impact of commonly used dietary emulsifiers on human gut microbiota. *Microbiome*. 2021;9(1):66. doi:10.1186/s40168-020-00996-6
27. Nickerson KP, Homer CR, Kessler SP, et al. The Dietary Polysaccharide Maltodextrin Promotes Salmonella Survival and Mucosal Colonization in Mice. Kufer TA, ed. *PLoS One*. 2014;9(7):e101789. doi:10.1371/journal.pone.0101789
28. Laudisi F, Di Fusco D, Dinallo V, et al. The Food Additive Maltodextrin Promotes Endoplasmic Reticulum Stress–Driven Mucus Depletion and Exacerbates Intestinal

Inflammation. *Cell Mol Gastroenterol Hepatol*. 2019;7(2):457-473. doi:10.1016/j.jcmgh.2018.09.002
29. Nickerson KP, McDonald C. Crohn's Disease-Associated Adherent-Invasive Escherichia coli Adhesion Is Enhanced by Exposure to the Ubiquitous Dietary Polysaccharide Maltodextrin. Mizoguchi E, ed. *PLoS One*. 2012;7(12):e52132. doi:10.1371/journal.pone.0052132
30. Wang S, Zhang S, Huang S, et al. Resistant Maltodextrin Alleviates Dextran Sulfate Sodium-Induced Intestinal Inflammatory Injury by Increasing Butyric Acid to Inhibit Proinflammatory Cytokine Levels. *Biomed Res Int*. 2020;2020:1-9. doi:10.1155/2020/7694734
31. Peter K, Rehli M, Singer K, Renner-Sattler K, Kreutz M. Lactic acid delays the inflammatory response of human monocytes. *Biochem Biophys Res Commun*. 2015;457(3):412-418. doi:10.1016/j.bbrc.2015.01.005
32. Ray SC, Baban B, Tucker MA, et al. Oral NaHCO3 Activates a Splenic Anti-Inflammatory Pathway: Evidence That Cholinergic Signals Are Transmitted via Mesothelial Cells. *The Journal of Immunology*. 2018;200(10):3568-3586. doi:10.4049/jimmunol.1701605
33. Sylvetsky AC, Clement RA, Stearrett N, et al. Consumption of sucralose- and acesulfame-potassium-containing diet soda alters the relative abundance of microbial taxa at the species level: findings of two pilot studies. *Applied Physiology, Nutrition, and Metabolism*. 2024;49(1):125-134. doi:10.1139/apnm-2022-0471
34. Hanawa Y, Higashiyama M, Kurihara C, et al. Acesulfame potassium induces dysbiosis and intestinal injury with enhanced lymphocyte migration to intestinal mucosa. *J Gastroenterol Hepatol*. 2021;36(11):3140-3148. doi:10.1111/jgh.15654
35. Tomassen MMM, Govers C, Vos AP, de Wit NJW. Dietary fat induced chylomicron-mediated LPS translocation in a bicameral Caco-2cell model. *Lipids Health Dis*. 2023;22(1):4. doi:10.1186/s12944-022-01754-3
36. Barnes EL, Nestor M, Onyewadume L, et al. High Dietary Intake of Specific Fatty Acids Increases Risk of Flares in Patients With Ulcerative Colitis in Remission During Treatment With Aminosalicylates. *Clinical Gastroenterology and Hepatology*. 2017;15(9):1390-1396.e1. doi:10.1016/j.cgh.2016.12.036
37. Yang F, Zhang S, Tian M, Chen J, Chen F, Guan W. Different Sources of High Fat Diet Induces Marked Changes in Gut Microbiota of Nursery Pigs. *Front Microbiol*. 2020;11. doi:10.3389/fmicb.2020.00859
38. Gori M, Altomare A, Cocca S, et al. Palmitic Acid Affects Intestinal Epithelial Barrier Integrity and Permeability In Vitro. *Antioxidants*. 2020;9(5):417. doi:10.3390/antiox9050417
